# Differences in Sexual Behavior of Teenagers and Young Adults with Cerebral Palsy: The Role of Sexual Needs and Sexual Esteem

**DOI:** 10.1007/s11195-017-9512-x

**Published:** 2017-11-29

**Authors:** Klaudia Czapla, Wojciech Otrębski

**Affiliations:** The John Paul II Catholic University of Lublin, Al. Racławickie 14, 20-950 Lublin, Poland

**Keywords:** Sexual behavior, Cerebral palsy, Youth and young adults, Poland

## Abstract

Sexuality is an inherent attribute of all human beings regardless of their race, religion and the level of physical fitness. The way it is perceived and manifested is determined by a number of biopsychosocial factors. In some people, including persons with cerebral palsy, the factors and their influence are rooted in the psychophysical condition of the human body. The aim of this study was to answer the question about how the levels of sexual esteem and sexual needs differentiate the sexual behaviors of young people with cerebral palsy. The study being presented was conducted with 62 young persons with cerebral palsy (half women and half men), who were selected using purposive sampling. They were aged 15–25 years and were individuals without cognitive difficulties. The research tool used was the Cerebral Palsy Individual’s Sexual Behavior Questionnaire developed by the authors. The findings of the study showed that half of the participants engaged in various sexual behaviors from the list that was presented to them. The frequencies of these engagements depended on the levels of their sexual esteem and sexual needs. Persons characterized by high levels of sexual esteem and sexual needs (much fewer than those with the low levels of both characteristics) engaged in sexual behaviors significantly more frequently. The most frequent among them was direct engagement in sexual activity (petting and sexual intercourse; *p* ≤ .001) and then exposure to sexually explicit magazines or films (*p* ≤ .05).

## Introduction

Human behavior is influenced by many different factors. There is motivation behind each decision people make [44, 45, 49–52, 55, 56]. Among the main factors that influence a behavior-regulation process, there are self-esteem and psychological needs. Human sexuality is a complex issue that as well as being an important aspect of our functioning is also a scientific discipline encompassing the psychology of emotional and motivational processes, and part of the model approximating the regulation of human functioning. The self-image of one’s sex life (sexual esteem) and sexual needs that all people develop guide their actions in this intimate sphere of life [7, 12, 61, 62, 66, 72]. However, sexual esteem and sexual needs are not the only factors that influence sexual activity manifested through specific sexual behaviors [21, 26, 49]: their range includes also body performance and especially the level of physical fitness. This means that motor disabilities can reduce or interfere with the possibility of having a satisfactory sex life that all people seek regardless of their race, beliefs or the level of fitness [5, 12, 13, 31, 32, 43].

According to a widespread view, human sexuality and sexual activity are directly associated with each other. In analyzing both these phenomena a number of crucial psychological factors, including sexual needs and sexual esteem, need to be considered.

Sexual needs and sexual esteem are part of broader mental concepts concerning psychological needs and global self-esteem. Each of them determine individuals’ everyday functioning, the psychological aspects of their sexuality, and the sexual activity in which they engage [53, 77, 86].

Sexual needs analyzed by researchers [51] are considered to be part of a greater whole called psychological needs. They can be defined as *biologically conditioned characteristics of the body, which manifest themselves as desire for sexual satisfaction through relieving recurring psychophysical tension through specific sexual activities that provide the opportunity for experiencing sensual pleasure.*

Because neither sexology nor psychology or other social sciences have well recognized sexual esteem [16, 35, 36], it is difficult to create a uniform definition explaining the issue of self-image as relating to erotic life. Having studied this personal characteristic in the late 1980s and early 1990s, Snell [68] defined it as *a generalized tendency to engage in nonspecific internal reinforcement toward oneself, as a result of one’s capacity to relate sexually to another person* (p. 256). He also concluded that sexual esteem creates erotic behavior towards other persons, but it is not correlated with autoerotic behavior [68]. Taleporos et al. [78] the authors of the PDSBE scale measuring the impact of disability on one’s body image and sexual esteem, view sexual esteem as *a positive regard for and confidence in an individual’s capacity to experience his or her sexuality in a satisfying and enjoyable way* (p. 177).

As a means of regulating individual’s relationships with the outer world and a reaction to internal and external stimuli, sexual behavior is similar in its function to behaviors displayed in relation to any other area of life [20, 22, 25]. Its purpose (sometimes unrealized) is to achieve sexual satisfaction [23–27]. Sexuality has more manifestations than sexual intercourse alone. Their range includes also petting, masturbation and interactions with the partner involving cuddling, kissing and dating [2].

The above outline of sexual needs and sexual esteem based on evidence from research leads us to the conclusion that they can influence a range of sexual behaviors. One of the question that arises from Kurpiel’s [37, 39, 70] findings is whether the extent of this influence on the intimate life of persons with cerebral palsy (CP) is similar or different from that observed among able-bodied persons.

Recent years have witnessed an increasing number of studies investigating human sexuality with respect to different levels of disability. Their main focus is on persons with intellectual disability or spinal cord injuries, mostly those who were sexually active when becoming disabled and wished to have a satisfactory sex life again [6, 9, 14, 33, 34, 36, 39–42, 63, 79, 80]. The difference between them and people with intellectual disabilities is that the latter need sexual education to understand what situations are likely to be perceived as embarrassing by other people, and as a protection from sexual abuse [8, 46–48, 54, 57, 59, 60].

Although the sexuality of people with CP is not different from that characterizing all other people, the literature does not explain this issue in detail. Most definitions of CP describe it as [39] *the impairment of the motor center in the brain, which occurs during pregnancy or childbirth, or in the perinatal period. This impairment is permanent and constant (non*-*progressive) in nature but due to its occurrence during brain development, it causes a number of motor dysfunctions* (p. 15). When serious, such dysfunctions can be a serious hindrance to having a satisfactory sex life [17].

The sexuality of persons with CP has not been sufficiently explored yet. Discussing primary sexual functions, Radomski [58, 59] notes that brain damage can impair the physical dysfunction, and consequently sexual activity, in persons with cerebral palsy, as well as raising the risk of cryptorchism, dysmenorrhea, and sexual dysfunctions (e.g. vaginismus). In many cases the sexual maturation of these persons starts earlier and lasts longer than in their individuals without CP [17, 58]. As far as secondary sexual functions are concerned, the sexuality of persons with CP is practically absent from the Polish literature. As for international studies, relatively few findings have been reported by Wiegerink [81–85], a leading researcher in this field. The focus of her studies is on the sexual activity of persons with CP in the context of their social relations. Her findings show that the CP patients go out on dates but their sexual activity starts at a later age than in the case of their able-bodied peers, that they are less likely to experience romantic relationships, and that they often face a lack of acceptance from others [82, 85]. At the same time, men with cerebral palsy who are in permanent relationships expect, and experience, sexual intimacy [64]. The results of studies into the sexual initiation of people suffering from CP are mutually exclusive and ambiguous. They either show that sexual intimacy is less frequent in this group than among their able-bodied peers, or lead to a conclusion that the two groups are not different in this respect [81].

CP does not prevent patients from engaging in autoerotic behavior, which is more frequent among men than among women [84]. According to Wiegierink et al. [83, 85], insufficient sexual education causes that persons with CP usually know less of sexuality than their able-bodied peers, and so they expect information on specific sexual problems associated with their disability [81]. Other researchers have found no differences in the sex life of persons with different types of cerebral palsy and have concluded that better motor skills of young people with CP increase the range of sexual behaviors [10]. Another finding has been that men tend to engage in autoerotic behavior more often than women do [13].

All the above studies reveal, regardless of how many years ago they were conducted, the likely problems of people with disabilities who wish to engage in sexual activity. At the same time, it is noticeable that the mainstream research excludes persons with cerebral palsy and that the issue of their sexual esteem and sexual needs still waits to be explored. Higher vulnerability of persons with disabilities to sexual violence makes it necessary to study their sexuality taking account of different disabilities [15, 29]. As the existing knowledge of their sexuality is poorly defined and rather limited, especially regarding persons with cerebral palsy, this and other research initiatives into this intimate area of human life seem to be necessary.

## Methodological Basis of the Study

The aim of this analysis was to determine how the levels of sexual esteem and sexual needs contribute to variations in the sexual behavior of young people with cerebral palsy. Earlier analyses and their findings published in our report [12] enabled us to dichotomize respondents into two extreme, statistically significantly different subgroups based on the co-presence of high levels and low levels of sexual esteem and sexual needs, respectively. The survey based on the sexual needs questionnaire led to the creation of respondents’ profiles encompassing different aspects of human sexual needs—the existence of such needs, a desire for sexual interaction, preferred frequency of sexual interactions. Because the last two elements were described by means of single-item scales complementing the main 9-item scale, there was no justification for using them in analysis as legitimate quantitative variables.

To make sure that no information on the existing sexual needs, desire for sexual interaction, preferred frequency of sexual interactions was omitted from analysis, a procedure analyzing all items from both tools (21 altogether) was applied. The procedure is described in detail in the previous publication [12].

### Subjects

The study was carried out with 62 subjects (M = 21.03 and SD = 2.70; half women and half men); (Table 1). One third of them were found to have high levels of sexual needs and sexual esteem (H-SN&SE) and in 2/3 the levels were low (L-SN&SE) (Table 2).

**Table 1:** Group characteristics

Age	M	SD
	21.03	2.70
Gender	Male (%)	Female (%)
	49.2	50.8

**Table 2:** Frequency (f) and percentage (%) of subjects as indicated by scales measuring sexual needs and sexual esteem by subgroup

Subgroup	f	%
High levels of sexual needs and sexual esteem (HLSN&SE)	22	35.50
Moderate levels of sexual esteem and sexual needs	1	1.60
Low levels of sexual needs and sexual esteem (LLSN&SE)	39	62.90

Subjects were selected using purposive sampling from among persons aged 15–25 years, who were diagnosed with cerebral palsy and were individuals without cognitive difficulties. This specific age band was selected for two reasons. One is legal in nature—the law sets the age of consent at 15 years. The second reason is that the transition from adolescence to adulthood takes place between the ages of 15 and 25; psychosexual development is then advanced enough to allow an individual to consciously engage in different sexual behaviors [1, 2].

### Methods

The research purpose was fulfilled by means of an individual survey and the Cerebral Palsy Individual’s Sexual Behavior Questionnaire (CPI-SBQ) that Czapla and Otrębski [11] developed from the revised Intellectually Disabled Individual’s Sexual Behavior Questionnaire authored by Czusz and Otrębski [14]. The questionnaire covers behaviors such as dating, cuddling, kissing, having a boyfriend/girlfriend, masturbating, petting, sexual initiation, sexual intercourses after initiation, and exposure to sexually explicit magazines and films. Each behavior was assessed for frequency and the subject’s emotions towards it.

The scores were analyzed to determine respondents’ sexual behaviors and their emotions about them. The Cronbach α coefficient for nine sexual behaviors in the studied population was .90.

## Results

### Manifestations of Sexual Behaviors in Young People with Cerebral Palsy

In order to determine how frequently subjects in subgroups H-SN&SE and L-SN&SE engaged in each of the nine sexual behaviors, they were asked to state the frequency of their sexual endeavors and their feelings towards them. The results were then analyzed and interpreted. An overview of this section can be found in Table 3. Tables 1 and 2 present the frequencies of sexual behaviors and the emotions associated with them for both subgroups.

**Table 3:** Percentage of subjects representing the dominant (prevalent) frequency (f) and emotions associated with particular sexual behaviors by subgroup

High levels of sexual esteem and sexual needs	Low levels of sexual esteem and sexual needs^a^
Dating: sometimes—59.10 Emotions: positive—86.50	Dating: never, sometimes—28.20 each Emotions (for sometimes): positive—89.00
Cuddling (with a boyfriend/girlfriend): sometimes—36.40 Emotions: positive—94.50	Cuddling (with a boyfriend/girlfriend): never—61.50 Emotions: positive—100.00
Kissing: sometimes—40.90 Emotions: positive—90.00	Kissing: never—28.20% Emotions: positive—93.40
Having a boyfriend/girlfriend: few relationships (2–3)—54.50 Emotions: positive—90.00	Having a boyfriend/girlfriend: never—48.70 Emotions: positive—90.00
Masturbation: very rarely—40.90 Emotions: positive—75.00	Masturbation: never—43.60 Emotions: positive—63.70
Petting: sometimes—54.50 Emotions: positive—100.00	Petting: never—65.50 Emotions: positive—87.40
Sexual initiation: after 20—50.00 Emotions: positive—47.50	Sexual initiation: no—71.80% Emotions: positive—45.50
Sexual intercourse after initiation: sometimes, very often—31.80 each Emotions: positive—94.50	Sexual intercourse after initiation: never—79.50 Emotions: positive—87.50
Reading/watching…: sometimes—45.50 Emotions: positive—68.50	Reading/watching…: never—38.50% Emotions: positive—56.00

^a^Emotions of subjects in the L-SN&SE subgroup who had engaged in these sexual behaviors

Most respondents in the H-SN&SE subgroup (nearly 60.00%) went out on dates ‘sometimes’. In the L-SN&SE subgroup, 28.20% had never had a date before and another 28.20% dated ‘sometimes’. Dating evoked positive emotions in 86.50% of respondents in the H-SN&SE subgroup. The same emotions were felt by 89.00% of those in the L-SN&SE subgroup who had already had a date (Table 3).

As far as cuddling is concerned, in the H-SN&SE subgroup 36.10% of respondents occasionally cuddled with a boyfriend or a girlfriend, as opposed to as many as 61.50% of respondents in the L-SN&SE subgroup who had never cuddled. Practically all respondents stated that they felt positive emotions about cuddling: 94.50% of subjects in the H-SN&SE subgroup and all subjects in the L-SN&SE subgroup who had ever been cuddled by a partner considered it a pleasant experience (Table 3).

Kissing was also reported more frequently in the H-SN&SE subgroup than in the L-SN&SE subgroup. In the first group, most respondents (40.90%) kissed ‘sometimes’, whereas in the L-SN&SE subgroup around 30.0% of respondents had never kissed. For all subjects in the H-SN&SE subgroup and for 93.40% of respondents in the L-SN&SE subgroup kissing was an emotionally positive act (Table 3).

Another manifestation of human sexuality considered in the study was having a boyfriend/girlfriend. In the H-SN&SE subgroup as many as 54.50% of subjects had already been in a few relationships (2–3), while in the L-SN&SE subgroup almost 48.70% of respondents had never had a boyfriend or a girlfriend. Those who had been in relationships had positive emotions about having a partner (90% of respondents in both subgroups) (Table 3).

In addition to sexual interactions with other people, the scope of analysis included also autoerotic behavior. The biggest percentage of respondents in the H-SN&SE subgroup (40.90%) stated that they masturbated very rarely, whereas in the L-SN&SE subgroup 43.60% of respondents had never masturbated. In both subgroups, most subjects associated masturbation with positive emotions. Even though it was rated negatively more often than other sexual behaviors considered in the study were, 75.00% of subjects in the H-SN&SE subgroup and 63.70% of those in the L-SN&SE subgroup had positive views on masturbation (Table 3).

Petting or kissing, holding and touching another person in a sexual way was experienced ‘sometimes’ by 54.50% of subjects in the H-SN&SE subgroup, but in the L-SN&SE subgroup 61.50% of subjects did not have that experience at all. All subjects in the H-SN&SE subgroup who had petting experience felt positive about it; no one viewed it as emotionally negative or neutral. In the L-SN&SE subgroup, positive emotions about petting were reported by 87.50% of those who experienced it (Table 3).

Half of respondents in the H-SN&SE subgroup had had their first sexual intercourse after 20 years of age, but in the L-SN&SE subgroup as many as 71.70% did not have their sexual initiation yet. Most respondents associated it with positive emotions. Interestingly, the between-group differences in the shares of those who felt so were not so big as before: only half of subjects in the H-SN&SE subgroup (47.60%) and in the L-SN&SE subgroup (45.40%) had pleasant memories of their first sexual intercourse (Table 3).

Almost 60.00% of respondents in the H-SN&SE subgroup who had experienced sexual initiation engaged in sexual intercourses ‘sometimes’ or ‘very frequently’ (31.80% for each answer). This contrasts with the L-SN&SE subgroup where nearly 4 in 5 of subjects had only one sexual intercourse. In both subgroups, most of those who had gone through sexual initiation considered sexual intercourse an emotionally positive experience. Almost all subjects in the H-SN&SE subgroup and nearly 90.00% of subjects in the L-SN&SE subgroup had positive memories of this act (Table 3).

Almost half of respondents in the H-SN&SE subgroup read or watched sexually explicit magazines and films ‘sometimes’. In the L-SN&SE subgroup, nearly 40.00% of respondents had never done that. This type of sexual behavior also evoked positive emotions in the respondents: in more than 2 in 3 in the H-SN&SE subgroup and in almost 60.00% of subjects in the L-SN&SE subgroup (Table 3).

Following the determination of differences in the most frequent sexual behaviors between study participants with high and low levels of sexual needs and sexual esteem, the differences were tested for statistical significance. To this end, as the statistical procedure required, the number of categories describing the frequency of engagement in each sexual behavior was reduced from five (*never*, *once*, *very seldom*, *sometimes*, and *very often*) to two, namely *Yes* and *No*. As a result, all ‘*never*’ answers’ were summed up as *No* and all affirmative answers, i.e. *once, very rarely, sometimes, very often*, were aggregated as *Yes*. This approach ensured compliance with the requirements of the Chi-squared distribution and allowed the differences between particular sexual behaviors displayed by participants with distinct levels of sexual needs and sexual esteem to be assessed for significance (Table 4).

**Table 4:** Differences in the sexual behaviors of young persons with cerebral palsy and different levels of sexual esteem and sexual needs

Sexual behavior	Sexual behavior	High levels of sexual esteem and sexual needs		Low levels of sexual esteem and sexual needs		Chi-squared distribution	
		f	P	F	P	X^2^	P
Dating	No	0	0.00	10	25.60	5.006	.025
	Yes	22	100.00	29	74.40		
Cuddling	No	4	18.20	24	61.50	10.648	.001
	Yes	18	81.80	15	38.50		
Kissing	No	0	0.00	11	28.20	5.782	.016
	Yes	22	100.00	28	71.80		
Having a boyfriend/girlfriend	No	2	9.10	19	48.50	9.784	.002
	Yes	20	90.90	20	51.30		
Masturbation	No	1	4.50	16	41.00	9.312	.002
	Yes	21	95.50	23	59.00		
Petting	No	1	4.50	24	61.50	18.889	.0001
	Yes	21	95.50	15	38.50		
Sexual initiation	No	1	4.50	28	71.80	25.506	.001
	Yes	21	95.50	11	28.20		
Sexual intercourse after initiation	No	4	18.20	31	79.50	21.616	.001
	Yes	18	81.80	8	20.50		
Reading/watching sexually explicit magazines/films	No	3	13.60	15	38.50	4.167	.041
	Yes	19	86.40	24	61.50		

Again, the first manifestation of sexuality that was subjected to analysis was dating. In the H-SN&SE subgroup all respondents had been on dates and almost 3 of 4 those in the L-SN&SE subgroup (the other 1 in 4 had not dated yet). Statistical analysis showed the between-group differences to be significantly in favor of the H-SN&SE subgroup (*p* ≤ .005) (Table 4).

More than 80.00% of respondents in the H-SN&SE subgroup had cuddled and nearly 20.00% had not. In the L-SN&SE subgroup, most respondents (over 60.00%) had never been cuddled by a boyfriend/girlfriend (*p* ≤ .01). Similarly, the analysis of kissing pointed out that this sexual behavior was more frequent in the H-SN&SE subgroup than in the L-SN&SE subgroup (*p* ≤ .05) (Table 4).

The two subgroups differed with regard to having a boyfriend/girlfriend, which was statistically significantly more frequent in the H-SN&SE subgroup (*p* ≤ .01), where almost all of respondents (more than 90.00%) had been in a relationship and only less than 10.00% had not had a partner before. In the L-SN&SE subgroup, the respective rates were slightly above 50% and slightly below 50.00% (Table 4).

Autoerotic behaviors, such as masturbation, were common to almost all subjects in the H-SN&SE subgroup (> 95.00%). In the L-SN&SE subgroup, almost 60.00% of subjects had masturbated before and slightly more than 40.00% had never done it. The between-group differences were statistically significant (at *p* ≤ .05) (Table 4).

The analysis of three sexual behaviors directly related to sexual activity (petting, sexual initiation and post-initiation sexual intercourse) revealed between-group differences in the favor of the H-SN&SE subgroup (*p* ≤ .001) (Table 4).

The subgroups were also different in the number of subjects reporting exposure to sexually explicit content. Although in both subgroups more respondents than not read/watched sexually explicit magazines/films, the percentage of such respondents was higher in the H-SN&SE subgroup than in the L-SN&SE subgroup (nearly 90.00 and 60.00%, respectively) (*p* ≤ .05) (Table 4).

To sum up, statistical analysis showed that young persons with cerebral palsy and different levels of sexual needs and sexual esteem differed significantly in the frequency with which they engaged in each of the nine sexual activities considered in the study. The frequencies are considerably higher for the H-SN&SE subgroup than for the L-SN&SE subgroup, but nonetheless respondents in both subgroups are similar in their emotional attitude towards the activities. As the qualitative analysis showed (see previous section), it was mostly positive in both subgroups.

## Discussion

The study was undertaken to investigate the differences in the sex life of young persons with CP with respect to selected psychological aspects of human sexuality.

The subjects were first tested for the levels of sexual esteem and needs and then two subgroups were formed of those who scored high and low on the tests, respectively H-SN&SE and L-SN&SE. The factors explaining why the first subgroup was considerably bigger than the second one are laid out in another publication on the above-mentioned aspects of sexuality [13]. In this study, the sexual-life variables were used with the sole purpose of identifying the two subgroups to compare and analyze them with respect to differences in the sexual behaviors of their members.

The differences in the frequencies with which the participants engaged in the nine sexual activities considered in the study were statistically significant in favor of the H-SN&SE subgroup. A probable source of the differences is positive correlations between sexual needs and the frequency of sexual intercourses, which are also reported by some studies on the able-bodied populations [76]. The latter also point to higher self-esteem in young, able-bodied girls who are sexually active [86]. Another plausible explanation can be sought in the fact that some young people with cerebral palsy tend to alienate themselves and break ties with their peers as they graduate, unlike their able-bodied peers who increase their sexual activity [18]. Adulthood is a very difficult period for persons with cerebral palsy because of disability-related limitations and problems associated with the dependency on the parents [82]. This observation seems particularly relevant to the case of subjects in the L-SN&SE subgroup; experiencing problems with becoming independent they tend to alienate themselves, which compromises their chances of developing close sexual relationships and engaging in sexual activities, especially as a form of interaction with other people.

Having established the common factor behind the differences in all these behaviors, let us consider them on a case-by-case basis. Dating was significantly more frequent in the H-SN&SE subgroup, likewise cuddling, kissing and having a boyfriend/girlfriend, probably because respondents in this subgroup have more social relations facilitating the establishment of intimate and sexual relationships. Another plausible explanation is that high sexual esteem accompanied by high levels of global self-esteem and self-efficacy helps them establish relations with other people and, consequently, have dates and develop relationships, etc. [19, 38]. Subjects in the L-SN&SE subgroup are more inclined to alienate themselves and the stereotypes they are inculcated with prevent them from seeking relations with others. As they do not go out on dates, they have much fewer chances to build relationships. Women with disabilities have difficulty finding a date and consequently a partner. All this significantly diminishes their self-esteem, including sexual esteem [62, 82].

The probable cause of the differences in the frequency of masturbation lies in the level of sexual needs rather than sexual esteem. This observation agrees with the conclusion of Snell and Papini [67–69], according to whom sexual esteem and autoerotic behavior are not correlated with each other. Studies have showed that CP hampers the ability to masturbate and that many disabled persons capable of only limited interaction with other people seek to satisfy their sexual needs through autoerotic behavior [58, 85]. In the present study, the frequency of masturbation in the L-SN&SE subgroup was considerably lower than that in the H-SN&SE subgroup, most probably because of the low level and suppression of sexual needs even with respect to autoerotic behavior [72, 73]. A similar mechanism may apply to reading/watching sexually explicit magazines/films, as both activities are usually accompanied by self-stimulation of the genitals (masturbation).

Let us now present the study findings regarding petting, sexual initiation and engagement in sexual intercourses after initiation. An important contribution to this field of research has been made by Szczerba [71, 72], who argues that at some point of life young people decide whether to become sexually active or to continue in abstinence. Unlike the individuals without CP who are only guided by their morals, people with motor disabilities are also concerned about the physical aspect of sexual activity. A frequent source of this concern is their poor knowledge of the limitations related to their disability (p. 517) [28]. Although the results of studies show that only the severe forms of CP cause major problems during a sexual intercourse [17], people with disabilities have low awareness of sexuality issues. This can discourage them from becoming sexually active [3, 10, 85] and make them reluctant or fearful of their sexuality, consequently reducing their sexual esteem and sexual needs. Such attitudes were reported more frequently by the H-SN&SE subgroup, probably because the sexual esteem and sexual needs of these respondents were consistently strong due to the operation of the aforementioned factors. A noteworthy fact is that the sexually active respondents (the H-SN&SE subgroup) had their initiation after they turned 20. This seems to show that persons with cerebral palsy start a sex life (including sexual intercourses) at later age than their able-bodied peers that have been shown by research to have their first sexual intercourse at an average age of 18.4 [1, 30, 65].

Lastly, the analysis focused on how the respondents felt about each of the nine sexual behaviors. The two subgroups were not found to be different in that respect, the reported feelings were mostly positive in both of them. It is possible that disabled women find their sex life satisfactory despite hip pain and other physical problems [4, 48]. Earlier studies show, however, that some behaviors, e.g. sexual initiation (probably because of the associated pain) and masturbation (which may be disapproved on religious or moral grounds) usually evoke stronger negative emotions than others [74, 75].

This research project is innovative in many respects, but its limitations have to be noted too. First, because the study was expected to involve some challenges, a relatively small sample of respondents was assembled (62 persons), which somewhat limited the range of analytical options. Further, as the unavailability of fully standardized methods prevented a wider use of the quantitative analysis, the research had to focus also on the qualitative results. Its methods were, however, carefully selected and developed to best describe and characterize the investigated aspects of human sexuality. This situation shows that in order to gain insight into the various aspects of human sexuality new psychological tools need to be created or the existing ones appropriately revised.

The project’s findings acknowledge that people with congenital disability suffering from cerebral palsy feel a need for intimacy. This challenges stereotypes frequently perpetuated by their loved ones that this condition deprives people of sexual needs and the desire to have a sex life. The findings justify also further research initiatives into the various aspects of sexuality of persons with this dysfunction.

There are at least two benefits that studies using the aforementioned set of variables to explore the sexuality of persons with cerebral palsy can deliver. First, being innovative and having a cognitive value, the studies considerably expand the theoretical knowledge of this problem. Secondly, their findings can be used practically to make the sexual education or rehabilitation of such persons more effective. These issues are sometimes presented in the international literature, but neither the specific research problem nor the method used to investigate the selected aspects of sexuality has been presented so far.
